# Cognitive proximity for innovation: Why matters? an applied analysis

**DOI:** 10.1371/journal.pone.0283557

**Published:** 2023-05-24

**Authors:** Eduardo Sánchez-García, Javier Martínez-Falcó, Bartolomé Marco-Lajara, Jakub Pizoń

**Affiliations:** 1 Faculty of Economic and Business Sciences, Department of Management, University of Alicante, Alicante, Spain; 2 Faculty of Management, Lublin University of Technology, Lublin, Poland; University of Lisbon: Universidade de Lisboa, PORTUGAL

## Abstract

The purpose of this research is to deepen the study of the influence of cognitive proximity has on the innovative performance of firms, as well as the mediating effect of potential and realized absorptive capacity in this relationship. For this purpose, an empirical analysis has been carried out. The primary data have been analyzed by means of PLS-SEM technique. The results show that the cognitive proximity of firms has both a direct and an indirect impact on their innovative performance, through their potential and realized absorptive capacity. We conclude that cognitive proximity matters for the innovation performance of firms, as it facilitates the understanding and establishment of positive reciprocity agreements between the companies, especially in terms of knowledge. Nevertheless, firms must develop a great capability to absorb new knowledge to exploit the advantages derived from its cognitive proximity to its stakeholders and leverage all the knowledge within their reach.

## Introduction

Social capital in its cognitive dimension relates to the perception and comprehension of the language, norms, codes, values, and objectives shared by the members of the social network, which, according to [1], promotes interaction between the network nodes and increases the network’s effectiveness. The cognitive component demonstrates how to build and cultivate productive social interactions within a specific network or social context [2]. Cognitive closeness pulls comparable organizations together in the densest places, hence increasing information sharing [3]. Therefore, access to knowledge might be contingent upon the degree of cognitive closeness between firms, affecting the chances of firms to access external valuable knowledge and using it to enhance their innovation performance [4,5].

Regional differences may impact the way in which organizations operate and how they innovate and share their knowledge. In this vein, external knowledge resources are increasingly seen as a crucial requirement for the success of businesses in a variety of domains, notably in terms of innovation, which is the most potent driver of socioeconomic development [6,7]. By engaging with other useful sources of knowledge, firms may absorb, integrate, and exploit new information [8]. In this manner, a broad knowledge base provides the basis for the growth of the absorptive capacity of firms [9]. This notion might be viewed as the firm’s capacity to recognize new information, acquire it, and utilize it in order to advance the innovative development of the organization [10–12]. This paper, however, considers four dimensions in this process, based on the work of Zahra and George [13], who conceptualized it by establishing four dimensions and grouping them in two blocks: acquisition and assimilation (potential absorptive capacity) and transformation and exploitation (as realized absorptive capacity), henceforth called PACAP and RACAP, respectively.

The leverage of external information sources is seen as one of the most essential processes behind organizations’ innovative operations [8,14,15]. Through the management of external information, organizations are able to produce value and obtain and maintain a competitive advantage through their absorptive capacity. Then, it may boost the rate and frequency of innovation inside a company [16]. Innovation has been shown to be connected to absorptive capacity via the single-step effect of PACAP on RACAP [17,18]. Despite this, several research have contradictory findings and then should be examined further [12,19,20]. Huang *et al*. [21] shows that absorptive capacity may spur business performance in terms of innovation. To the best of our knowledge, however, no research combines the variables of PACAP and RACAP to examine their involvement in the link between cognitive proximity and innovative performance of energy firms. To fill this void, we examine the serial mediation impact of these dimensions on the established relationship. This work complements research in adjacent fields and offers a novel research approach for future investigations. The preceding discussion raises the topic of whether proximity in cognitive terms influences innovative performance. Both levels of absorptive capacity are potential mediators of this association.

The purpose of this research is to empirically analyze the effect of cognitive proximity on the innovative performance of enterprises in the sector under investigation, as well as the mediation effect of the two dimensions of absorptive capacity detailed above. Then this research adds to the literature by presenting empirical data about the relevance for companies to interact and collaborate with those agents with whom they maintain cognitive ties, which facilitate communication and collaboration in favor of the achievement of shared objectives, having a positive and significant effect on the identification, assimilation, transformation, and exploitation of knowledge and the innovative performance of firms. Thus, this paper examines the importance of the cognitive proximity of firms as a driver of the absorptive capacity of companies for innovative purposes.

The analysis is carried out as outlined below. A questionnaire including valid and reliable scales for estimating the variables contained in the suggested model was developed and administered to the companies examined, generating 197 valid units. PLS-SEM was used to estimate the hypothesized relationship. Replies were uniquely tagged and evaluated using SmartPLS software version 3.9.

The research is structured as described below. The research hypotheses and model nomogram are derived from a literature review pertinent to the investigated variables and relationships. The methodology of the investigation is then described, followed by its findings. The study’s conclusion emphasizes the need for more research into the impacts of social capital and absorptive capacity on corporate innovation.

## Literature review

### Cognitive social capital and innovative performance

Firms tend to share qualities such as language, conventions, and legal frameworks, which build and maintain local trust [22,23]. Small companies, which make up most of the business structure of the countries, are crucial for the diffusion of innovations because they rely more on localized networks and informal communication, capitalizing on the tacit knowledge exchanges that occur locally [24,25]. Innovation is regarded from an evolutionary standpoint as an unpredictable and cumulative process [26]. As a strategy of risk reduction, firms may reduce the uncertainty of innovation by conducting an external scan to identify and obtain external knowledge inputs. Integration of external data is a complex and demanding procedure, partly due to the fuzziness of the borders between distinct kinds of knowledge and technologies, which makes it difficult for businesses to successfully look for external knowledge inputs [27]. A company’s ability for innovation is constrained not just by its boundaries, but also by the cognitive proximity achieved in particular places. Some empirical research has revealed cognitive proximity as the main mechanism for the formation of informal knowledge-circulating networks, and then suggesting that innovation outcomes and inter-organizational learning require the existence of social and cognitive proximity among firms [28–30].

According to empirical research, spillovers among cognitive close firms contribute to increase their economic and innovation performance [31,32]. On the basis of shared or comparable cognitive patterns and values, communication is often vigorous and fruitful. Consensus is reached by integrating the differences between the parties and engaging in cooperative sets. Through the process of achieving agreement, the cognitive pattern of the opposing stakeholder collides with the existing firm’s cognition and breaks its initial cognitive boundary [33]. Afterwards, a company with a novel cognitive pattern may seek diverse information through the process of interaction, fostering corporate innovation and serving as a point of innovation diffusion [34]. Furthermore, cognitive social capital is shaped through firms shared long-term declarations, objectives, and values [35]. Therefore, this factor is crucial for firms to overcome various constraints [36]. Previous research has shown that social capital facilitates firm innovation in an environment that is reasonably stable [37,38]. Nevertheless, the present dynamism of the environment necessitates a more in-depth examination of the elements within the control of businesses that drive their creative success.

In terms of the connection of the mentioned variables, bonds between firms based on shared values or consensus might enhance the flow of knowledge and data [39]. Then, it is rational to assume that cognitive social capital has significantly contributed to the increase of innovative performance of organizations. Considering the above, the following hypothesis is proposed:

*Hypothesis 1 (+)*: There is a positive and significant relationship between cognitive proximity and the innovative performance of firms.

### Potential absorptive capacity

Making the difference between PACAP and RACAP allows researchers to investigate why some organizations fail due to changes in their external settings, such as technology or industry development, while others prosper under the same circumstances [13]. In this respect, a company’s ability to detect and incorporate value for commercial reasons seems to be crucial [11]. From an innovation-oriented perspective, absorptive capacity prompted a new conversation in management from the start [40,41]. Initially, it was seen as the external value recognition of information to be implemented in business, but over time, numerous other perspectives that were more focused on innovation emerged [11,42]. According to Zahra & George [13], acquisition is the capacity to recognize and acquire external information, while assimilation is its interpretation and comprehension. PACAP is the first stage of absorptive capacity, during which companies acquire external information and assimilate it.

Assuming that such information is relevant to the firm’s main business, each component may affect innovation [12]. Thus, PACAP promotes innovation by offering a flexible approach that allows businesses to modify and reconfigure organizational activities [43]. This is especially prominent in managing organizations with technology and the capacity to conform and adapt to relevant external information [18]. Firms with a robust PACAP can absorb the outcomes of obtaining new information and then, have the possibility of mixing it with prior knowledge for the innovation process [20]. Consequently, the skill component of the organizational team will affect the PACAP and the capacity to innovate and achieve innovation success.

If knowledge is seen as a system of cognitive schemata, its assimilation implies that the capacity to learn relies on the knowledge base possessed in the region where occurs [44]. The similarity between the company’s and its partner’s knowledge bases could impact the firm’s capacity to absorb information stored by the partner firm. Similarity between businesses’ knowledge bases may boost interorganizational learning and firms’ innovation performance [45–47].

Then, firms may concentrate on widening their knowledge base by learning and absorbing new information from other companies in cognitive proximity and, as a result, leveraging their PACAP [48,49]. Therefore, PACAP may have a moderating influence on the relationship between the cognitive proximity of firms and their innovative performance. Consequently, the following hypothesis is proposed:

*Hypothesis 2 (+)*: Firms’ potential absorptive capacity exerts a mediating effect on the relationship between cognitive proximity and the innovative performance of firms.

### Innovative performance through cognitive proximity, and potential and realized absorptive capacity

Over time, absorptive capacity has generated varied viewpoints in the management discourse [40,41,50]. Although was first seen as the external value recognition of information to be implemented in business [11], this perception shifted over time because of numerous alternative perspectives that were more focused on innovation [42]. Among the views that broadened the notion is the separation of an organization’s potential from its routines and procedures [13]. Then, the theory acknowledges a number of research that address the two components of absorptive capacity jointly to produce RACAP, while others, such as Flatten *et al*. [51], even referred to a scale development for its confirmation. Due to the existence of this confirmation, the theory recognizes that absorptive capacity dimensions or components might be sequential, but also complimentary, or overlap via processes and routines. RACAP refers to the transformation and use of external knowledge [13]. New information may be a significant impetus for change and organizational progress, prompting businesses to decide how to increase their absorptive capacity [42,48,52].

By mixing old and new knowledge in a productive manner, novel correlations and connections between various information flows develop. This might lead to fresh views on how to enhance present operations or enter new markets in a distinctive manner. While the former might result in product innovation initiatives, the latter can result in market or process innovations [53]. Lastly, application refers to a company’s capacity to commercially use new external information to fulfill organizational objectives, incorporating both market and technology knowledge [45,54]. Market knowledge informs businesses on how to market their expertise, while technical knowledge reveals how to build new production processes [55]. Thus, the intended consequence of absorptive capacity is the commercial application of new knowledge [56].

More recently, it has been acknowledged in the literature that this notion has branched out into numerous dimensions that distinguish its two aspects PACAP and RACAP [17,20]. This second kind demonstrates that innovation as a competitive advantage has an influence that narrows the gap between the two types of absorptive capacity [12,57]. RACAP is a measure of an organization’s internal efforts to apply acquired knowledge. PACAP may impact RACAP and develop dynamic organizational capacities, serving as an indication of knowledge generation [13,58].

**Fig 1:**
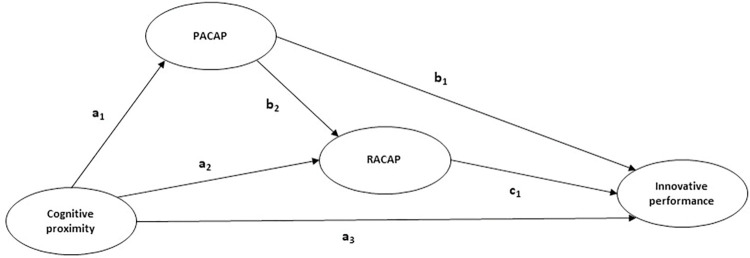
Nomogram of the proposed model. H1 = a3: Cognitive proximity → Innovative performance. H2 = a1 x b1: Cognitive proximity → PACAP → Innovative performance. H3 = a2 x c1: Cognitive proximity → RACAP → Innovative performance. H4 = a1 x b2 x c1: Cognitive proximity → PACAP → RACAP → Innovative performance.

PACAP also decreases the relative gap that may exist between the two kinds of capabilities in relation to the detection of innovation trends [57]. Once a business identifies a structural single mode pattern to innovate and learn and induces a contingency approach to management in order to provide value, it gains and maintains a competitive advantage [59,60]. Firms must enhance both aspects of their absorptive capacity to gain from the advantages of their cognitive proximity, using the external knowledge within their reach to their advantage in terms of innovation. Given the above, are proposed hypotheses 3 and 4. Furthermore, in Fig 1 is displayed the nomogram of the proposed model.

*Hypothesis 3 (+)*: Firms’ realized absorptive capacity exerts a mediating effect on the relationship between cognitive proximity and the innovative performance of firms.

*Hypothesis 4 (+)*: There is a double mediation of the potential and realized absorptive capacity in the relationship between cognitive proximity and the innovative performance of firms.

## Methodology

### Population and sample

The population under examination consists of Spanish enterprises operating in the domain of power supply. According to the SABI database, in 2019 there were 13,339 firms functioning in Spain. The sample includes 197 operational Spanish businesses. Despite employing just 2% of the overall workforce in Spain, this sector contributed 13.8% of the gross added value and 9.4% of industrial output in 2019, making it the second most significant sector. Moreover, was the sector with the highest employee productivity (466,500 euros on average).

### Data collection and measurement of variables

By creating and distributing a questionnaire data were collected. After evaluating the statistical validity of the completed surveys and deleting those deemed invalid (due to a substantial amount of lost data, patterns of response, or single-value responses), 197 valid replies were obtained. Through their "minimum R^2^" technique, Hair *et al*. [61] show that a model with a minimum R^2^ value of 0.500 and a maximum of two predictors requires a minimum sample size of 33 instances.

Cognitive social capital (independent variable). Is a seven-point Likert scale and has seven components. Is based on the dimension developed by Nahapiet and Ghoshal [36] and used by Parra-Requena *et al*. [62].

Innovative performance (dependent variable) was evaluated using a seven-point, thirteen-item scale. On the basis of the study of Prajogo and Ahmed [63] and Škerlavaj *et al*. [64], validated scales comprised of five, four, three, and one items were used to assess the innovative performance of the product, process, marketing, and management, respectively.

PACAP and RACAP (mediating variables): seven items make up each one of these variables (PACAP: acquisition, assimilation) and (RACAP: transformation, and exploitation) based on the work of Zahra and George [13] and Flatten *et al*. [51], being used a 7-point Likert scale.

### Analysis technique

To evaluate the hypotheses, we used the multivariate second-generation partial least squares, the PLS-SEM technique, a multivariate analytical approach. A great number of researchers in the field of strategic business management have put their focus on this technique [65]. In this instance, version 3.9 of SmartPLS was employed. According to Hair *et al*., [66], this method is appropriate for predictive analytics, particularly in the social sciences, due to the latent character of the variables considered in this field.

## Results

### Measurement model assessment

To assess the measurement model, it must be investigated its internal consistency, convergent validity, and discriminant validity [66]. By analyzing internal consistency reliability, the aim is to determine that the indicators of a latent variable measure the same construct, and their degree of correlation. Cronbach’s alpha [α] has traditionally been the instrument used to evaluate internal consistency. However, due to its sensitivity in relation to the number of items in the measurement scale and its tendency to underestimate the reliability of internal consistency, it is considered an excessively conservative measure [66]. These authors recommend its use combined with composite reliability [ρc], as the latter tends, on the contrary, to overestimate internal consistency reliability, so that the actual reliability would be in the range determined by both values. Moreover, unlike Cronbach’s alpha, composite reliability does not assume an equal distribution of the weighting of the indicators [67]. There is a third method, called Dijkstra-Henseler’s rho [ρA], which is considered as a measure of consistent reliability [68]. Then, Dijkstra-Henseler’s rho (ρA) is utilized, according to these authors. As seen in Table 1, and according to Chin [67], Dijkstra and Henseler [68], and Hair *et al*. [66], data presents a good internal consistency, as the value of Dijkstra-Henseler’s rho (ρA) is greater than 0.7.

**Table 1:** Assessment of internal consistency and convergent validity.

**INTERNAL CONSISTENCY**			**CONVERGENT VALIDITY**	
** **	**Dijkstra-Henseler’s rho (ρA)**		**Average extracted variance (AVE)**	
**Cognitive proximity**	0.872		0.565	
**Innovative performance**	0.851		0.689	
**PACAP**	0.702		0.759	
**RACAP**	0.751		0.788	
**CONVERGENT VALIDITY**				
**OUTER LOADINGS (λ)**	**C.P.**	**I.P.**	**PACAP**	**RACAP**
**CA adquisición**			0.844	
**CA asimilación**			0.897	
**CA explotación**				0.909
**CA transformación**				0.866
**DI gestión**		0.817		
**DI marketing**		0.787		
**DI proceso**		0.857		
**DI producto**		0.857		
**C.P. 1**	0.716			
**C.P. 2**	0.737			
**C.P. 3**	0.759			
**C.P. 4**	0.759			
**C.P. 5**	0.729			
**C.P. 6**	0.793			
**C.P. 7**	0.766			
**VIF**	**C.P.**	**I.P.**	**PACAP**	**RACAP**
**Cognitive proximity**		1.674	1.000	1.439
**PACAP**		1.977		1.439
**RACAP**		2.167		

Source: Own elaboration.

Note: C.P.: Cognitive proximity; PACAP: Potential absorptive capacity; RACAP: Realized absorptive capacity; I.P.: Innovative performance.

Convergent validity analysis establishes the intensity of the positive correlation between indicators measuring the same construct. To meet this requirement, the indicators must share a high proportion of their variance. To confirm convergent validity, the measurement is performed by evaluating the reliability of the indicators, i.e., the size of the outer loadings (λ), and the Average Variance Extracted (AVE), which refers to the total mean value of the squared loadings of the indicators belonging to the same construct [66]. After evaluating the results shown in Table 1, it can be confirmed that the loadings of the indicators are at adequate levels of individual reliability. Likewise, the value of the AVE of the constructs is considerably higher than the established minimum values (the outer loadings have a value more than 0.707 and the AVE is higher than 0.5). Therefore, this requirement is also met, according to Henseler *et al*. [69] and Hair *et al*. [66].

Regarding to the variance inflation factor (VIF), data show no multicollinearity, as all VIF values are less than 3 [70].

The purpose of discriminant validity assessment is to determine the degree to which each construct is unique, i.e., that it captures phenomena that are distinct from the rest of the constructs that make up the model. Historically, cross-loading analysis and the Fornell and Larcker method have been used. However, the Heterotrait-Monotrait Ratio (HTMT) is a more effective tool for determining discriminant validity difficulties [69]. Kline [71] states that the HTMT ratio must be less than 0.85, which indicates that all constructs are empirically different. The model largely satisfies this criterion, as demonstrated by Table 2.

**Table 2:** Evaluation of discriminant validity.

DISCRIMINANT VALIDITY				
HTMT	C.P.	I.P.	PACAP	RACAP
**Innovative performance**	0.681			
**PACAP**	0.708	0.678		
**RACAP**	0.739	0.809	0.827	

Source: Own elaboration.

Note: C.P.: Cognitive proximity; PACAP: Potential absorptive capacity; RACAP: Realized absorptive capacity; I.P.: Innovative performance.

### Structural model assessment

The evaluation of the structural model helps us to determine the model’s predictive power and the nature of the model’s numerous latent variables’ interrelationships, and so to evaluate the hypotheses provided within the theoretical framework. The evaluation of the structural model is undertaken in accordance with the method outlined by Hair *et al*. [66]. In the first step, an Algorithm PLS analysis is performed to assess the degree of collinearity between the predicted constructs, with the VIF value kept below 3 [70].

The path coefficients of the established associations are then calculated by executing the bootstrapping procedure in full mode with 5000 random subsamples and a 99% confidence interval. These coefficients, whose values range from 0 to 1, reflect the extent to which a change in the value of the source variable affects the value of the target variable. The R^2^ coefficients are then used to evaluate the predictive power of the model for each variable. According to Hair *et al*. [66], R^2^ values of 0.25, 0.50, and 0.75 are weak, moderate, and significant, respectively. Next, the ƒ^2^ size of the effects is analyzed to assess the influence of each exogenous construct on the R^2^ value of the related endogenous latent variable. If the ƒ^2^ value is close to 0.02, 0.15, or 0.35, it is classified as small, moderate, or large effect [66]. Lastly, the blindfolding method is utilized to examine the cross-validation redundancy index Q^2^, which reflects the predictive significance of the model with respect to each endogenous component. Q^2^ values greater than zero, 0.25, and 0.50, respectively, indicate low, moderate, and substantial predictive significance [72].

In the subsequent analysis, the omission distance D was determined by the constraint that the sample size cannot be divided by this number to yield an integer. Consequently, the D value selected was 7 [Sample size = 197]. According to Hair *et al*. [66], the significance and importance of the relationships, collinearity, the value of the coefficients of determination (R^2^), effect size (ƒ^2^), and predictive significance (Q^2^) must be evaluated. The direct and indirect effects of doing the bootstrapping technique in full mode with 5,000 random subsamples are shown in Tables 3 and 4, respectively.

**Table 3:** Summary of direct effects.

Structural path	Coefficient (β)	S.D.	P-values	95% CI	Results
C.P. -> I.P.	0.299**	0.073	0.000	[0.160–0.438]**	H1 supported
C.P. -> PACAP	0.552**	0.054	0.000	[0.448–0.646]**	
C.P. -> RACAP	0.330**	0.068	0.000	[0.200–0.462]**	
PACAP -> I.P.	0.087	0.074	0.243	[0.065–0.229]	
PACAP -> RACAP	0.499**	0.062	0.000	[0.376–0.622]**	
RACAP -> I.P.	0.400**	0.080	0.000	[0.237–0.547]**	

Source: Own elaboration.

Note: Coef.: Coefficient; S.D.: Standard deviation; C.I.: Confidence interval; C.P.: Cognitive proximity; I.P.: Innovative performance; PACAP: Potential absorptive capacity; RACAP: Realized absorptive capacity

** Statistically significant at 1%.

**Table 4:** Summary of indirect effects.

Total effect of C.P. on I.P.		Direct effect of C.P. on I.P.		Indirect effect of C.P. on I.P.			Conclusion
**Coef. (β)**	**T value**	**Coef. (β)**	**T value**	**Point estimated**		**I.C. 99%.**	
0.589**	**10.973**	0.299**	**4.110**	**Total**	**0.290**		
				H2 = a_1_ x b _1_	0.048	[-0.039–0.127]	H2 unsupported
				H3 = a_2_ x c _1_	0.132**	[0.062–0.205]	H3 supported
				H4 = a_1_ x b_2_ x c_1_	0.110**	[0.056–0.177]	H4 supported

Source: Own elaboration.

Note: Coef.: Coefficient; C.I.: Confidence interval; C.P.: Cognitive proximity; I.P.: Innovative performance

** Statistically significant at 1%.

Cognitive proximity has a positive and statistically significant effect on the innovative performance of firms [0.299, p = 0.000]. In addition, the variable RACAP mediate a positive and statistically significant indirect effect in this relationship [0.132, p = 0.000], although the variable PACAP has a positive but not significant mediation effect. Nevertheless, they both exerts a double mediation effect [0.110, p = 0.000], so that the capacity of companies to effectively transform and exploit knowledge is established as a key element for the development of innovations. The proposed model explains 30.5%, 53,9%, and 47,7% of the variance of the "PACAP" "RACAP" and "Innovative Performance" components, respectively.

The contribution of the exogenous construct "Cognitive proximity" to the determination coefficient of the endogenous latent variable "PACAP", "Innovative Performance" and "RACAP" (ƒ^2^) is moderate [0.102, 0.164] and high [0.439] respectively, and PACAP has a large ƒ^2^ effect over RACAP [0.374] [73]. Finally, the Q^2^ values of the endogenous variables’ “PACAP”, “RACAP”, and “Innovative Performance” are 0.221, 0.414 and 0.321, respectively, which indicates that the model has a moderate predictive relevance on the mentioned variables [72]. Then the three out of four hypotheses proposed are accepted.

## Discussion and conclusions

The impact of cognitive proximity on the absorptive capacity of firms have received little attention in the scientific literature. Particularly, the impacts of the cognitive proximity, which in theory seems to be a significant predictor of absorptive capacity development, have not been experimentally examined [42,74,75]. Furthermore, the theoretical outcomes of absorptive capacity have not been well supported by actual evidence. This study adds in a variety of ways to the study of internal and external variables that encourage organizations’ innovative performance. Hypothesis 1 is supported by our findings that the degree of cognitive proximity influences the innovation performance of enterprises. In addition, hypotheses 2, 3, and 4 demonstrate that the cognitive proximity of firms influence their absorptive capacity and, therefore, the way they harness new external knowledge when producing innovations. On the other hand, a positive but not significant impact of PACAP on the innovative performance of companies has been identified. This demonstrates that the discovery and acquisition of new information do not impact the growth of innovations in businesses; rather, it is the successful transformation and application of such knowledge that has this effect. In accordance with Xie *et al*. [76], it has been shown that RACAP mediates the link between the PACAP and the innovative performance of firms, indicating that knowledge transformation and exploitation are essential for firms to generate innovations successfully.

Although it has not been feasible to establish if the identification and acquisition of new knowledge has a positive and substantial influence on the innovative performance of businesses, it has been shown that this process happens via the effective use of RACAP. Similarly, Song *et al*. [42] claim that the base of absorbed knowledge does not play a direct role in firms’ innovativeness, but rather the use of this knowledge in value creation activities. It involves not just acquiring external information, but also applying it. The cognitive proximity seems to be a relevant aspect that has a direct effect on the innovative performance of businesses. Then, our results indicate that the cognitive characteristics of the firms foster their innovative performance both directly, due to the similarity of their knowledge base, and indirectly, through the investment of firms in enhancing their capacity to acquire and assimilate the new external knowledge, which is a crucial factor when conducting innovative activities. This research concludes that cognitive proximity favors the development of knowledge absorption skills, specially, those related with the effective application of new knowledge for innovative purposes and then, the improvement of their innovation performance.

The above has important managerial, political, and theoretical implications. With respect to the first ones, managers must be aware of the impact that cognitive features can have on firms’ performance, especially in terms of innovation. However, the fact of having cognitive proximity with the stakeholders of the firm does not guarantee the effective harnessing of the advantages that this implies. Thus, among other relevant aspects, managers must invest considerable efforts and resources in developing a great capacity to identify and assimilate as much valuable external knowledge as possible, because through it they can boost their performance in a multitude of areas, especially in terms of innovation. However, there is no point in acquiring knowledge if resources and effort are not put into applying it effectively. This last step has been shown to be fundamental in order to take advantage of the new external knowledge acquired by companies. In addition, in relation to the political implications, politicians must be aware of the importance of the cognitive proximity between firms when creating their territorial development policies, besides the well-known geographical proximity. However, they should not forget to establish mechanisms to help companies effectively absorb as much knowledge as possible, otherwise the benefits derived from the development strategies developed by the government would not be maximized. This could even lead leaders to think that their development strategies focused on geographical and cognitive proximity do not work, when the problem could be the difficulties faced by companies in internalizing and exploiting all the knowledge generated.

Finally, regarding to the theoretical implications, through cognitive proximity companies have access to a greater extent to valuable resources, particularly new knowledge. This study demonstrates the significance of firms’ cognitive features and their ability to absorb the external new knowledge as innovation drivers for firms. Then, this paper has important implications for the debate about what are the main internal features and capabilities of the firms to drive innovative performance, especially those mechanisms that favor knowledge diffusion between firms. In addition, it is increased the knowledge of how both PACAP and RACAP contribute to the innovative performance of businesses close in cognitive terms. These results provide insight into the importance of companies’ internal capabilities to take advantage of opportunities in their environment. Firms must develop their capacity to identify and assimilate new external knowledge but must also integrate all this knowledge into the firms’ knowledge base, transform it and apply it effectively for innovative purposes in order to have a real impact on their innovative performance. The results of this empirical analysis indicate that the effect of cognitive proximity on the process of businesses’ knowledge absorption capabilities with innovative purposes is a viable field for further study.

## Supporting information

S1 Data (XLSX)
